# Tumoral BRD4 expression in lymph node-negative breast cancer: association with T-bet+ tumor-infiltrating lymphocytes and disease-free survival

**DOI:** 10.1186/s12885-018-4653-6

**Published:** 2018-07-20

**Authors:** Minji Lee, Farnoosh Tayyari, Dushanthi Pinnaduwage, Jane Bayani, John M. S. Bartlett, Anna Marie Mulligan, Shelley B. Bull, Irene L. Andrulis

**Affiliations:** 1 Department of Laboratory Medicine & Pathobiology, University of Toronto, Toronto, ON Canada; 2 Laboratory Medicine Program, University Health Network, Toronto, ON Canada; 3 Fred A. Litwin Centre for Cancer Genetics, Lunenfeld-Tanenbaum Research Institute, Sinai Health System, 600 University Avenue, Toronto, ON M5G 1X5 Canada; 4 Ontario Institute for Cancer Research, Toronto, ON Canada; 5 Dalla Lana School of Public Health, University of Toronto, Toronto, ON Canada; 6 Department of Molecular Genetics, University of Toronto, Toronto, ON Canada; 7 Department of Pathology & Laboratory Medicine, Sinai Health System, Toronto, ON Canada

**Keywords:** Breast cancer, BRD4, Inflammation, TILs, Lymphocytic infiltration, T-bet

## Abstract

**Background:**

We previously observed that T-bet+ tumor-infiltrating T lymphocytes (T-bet+ TILs) in primary breast tumors were associated with adverse clinicopathological features, yet favorable clinical outcome. We identified BRD4 (Bromodomain-Containing Protein 4), a member of the  Bromodomain and Extra Terminal domain (BET) family, as a gene that distinguished T-bet+/high and T-bet−/low tumors. In clinical studies, BET inhibitors have been shown to suppress inflammation in various cancers, suggesting a potential link between BRD4 and immune infiltration in cancer. Hence, we examined the BRD4 expression and clinicopathological features of breast cancer.

**Methods:**

The cohort consisted of a prospectively ascertained consecutive series of women with axillary node-negative breast cancer with long follow-up. Gene expression microarray data were used to detect mRNAs differentially expressed between T-bet+/high (*n* = 6) and T-bet−/low (*n* = 41) tumors. Tissue microarrays (TMAs) constructed from tumors of 612 women were used to quantify expression of BRD4 by immunohistochemistry, which was analyzed for its association with T-bet+ TILs, Jagged1, clinicopathological features, and disease-free survival.

**Results:**

Microarray analysis indicated that BRD4 mRNA expression was up to 44-fold higher in T-bet+/high tumors compared to T-bet−/low tumors (*p* = 5.38E-05). Immunohistochemical expression of BRD4 in cancer cells was also shown to be associated with T-bet+ TILs (*p* = 0.0415) as well as with Jagged1 mRNA and protein expression (*p* = 0.0171, 0.0010 respectively). BRD4 expression correlated with larger tumor size (*p* = 0.0049), pre-menopausal status (*p* = 0.0018), and high Ki-67 proliferative index (*p* = 0.0009). Women with high tumoral BRD4 expression in the absence of T-bet+ TILs exhibited a significantly poorer outcome (log rank test *p* = 0.0165) relative to other subgroups.

**Conclusions:**

The association of BRD4 expression with T-bet+ TILs, and T-bet+ TIL-dependent disease-free survival suggests a potential link between BRD4-mediated tumor development and tumor immune surveillance, possibly through BRD4’s regulation of Jagged1 signaling pathways. Further understanding BRD4’s role in different immune contexts may help to identify an appropriate subset of breast cancer patients who may benefit from BET inhibitors without the risk of diminishing the anti-tumoral immune activity.

## Background

BRD4 (Bromodomain-Containing Protein 4) is a transcriptional epigenetic regulator that plays a crucial role in cancer and inflammatory diseases [1]. It is a member of the BET (Bromodomain and Extra Terminal domain) family that utilizes tandem bromodomains to recognize specific acetylated lysine residues in the N-terminal tails of histone proteins [2]. Upon interaction with chromatin, BRD4 has been shown to promote acetylation-dependent assembly of transcriptional regulator complexes that activate various transcriptional programs, such as those involved in cell proliferation and cell cycle control [3, 4].

Small molecule inhibitors that specifically target BET proteins have been demonstrated to interfere with expression of genes involved in cell growth and apoptosis evasion. Therapeutic benefits of the BET inhibitors have been observed in B-cell lymphoma [5] and acute myeloid leukemia [6, 7], as well as in lung [8], prostate [9], pancreatic [10], colorectal [11] and breast cancers [12]. Interestingly, BET inhibitors have also been shown to have an anti-inflammatory effect in the treatment of various inflammatory diseases and cancer [1, 13, 14], suggesting that BRD4 may have an active role in supporting inflammation.

Numerous studies have shown BRD4 to be important in the promotion of NF-kB-mediated transcription of inflammatory genes [15–17], whose functions in cancer initiation and progression have shown to be manifold and complex [18, 19]. Considering the clinical benefits of cancer immunotherapies that have been demonstrated through blockades of immune inhibitory pathways and stimulation of immune effector functions in tumors, investigating the potential link between BRD4 and immune infiltration in cancer may present a novel insight into the regulatory role of BRD4 in tumor immune surveillance.

Breast cancer is a complex and heterogeneous disease. Despite improvements in disease classification using tumor-related prognostic markers, a large disparity of clinical outcomes continues to be seen. This reflects the limitation of utilizing intrinsic tumoral characteristics as the sole determining factors of disease progression. An increasing number of studies have demonstrated that the components of tumor microenvironment, including immune infiltration, interact dynamically with the tumor, and influence clinical outcome. Particularly, infiltration by T lymphocytes has been shown to be associated with a good prognosis in breast cancer patients, and higher response rate to neoadjuvant therapy [20–27].

In two independent cohorts of women with familial breast cancer [28] and axillary node-negative (ANN) breast cancer [29], we have observed that T-bet+ tumor-infiltrating T lymphocytes (T-bet+ TILs) were associated with adverse clinicopathological features such as large tumor size, high grade, mutant p53, ER negativity, CK5 positivity, EGFR positivity, and basal molecular subtype [29, 30]. Despite being associated with an aggressive tumor phenotype, patients with a high level of T-bet+ TILs in their tumors had a favorable clinical outcome [29, 30]. T-bet is an immune-specific member of the T box family of transcription factors that is essential for differentiation of type 1 helper (Th1) T lymphocytes, as well as production of IFNy in CD4+ Th1 T lymphocytes and CD8+ cytotoxic T lymphocytes – subsets of immune cells that promote anti-tumoral inflammatory response [31, 32].

To examine how T-bet+ TILs may be associated with tumor development, we further investigated gene expression differences associated with T-bet+ TILs, and assessed their clinicopathological implications. Here we show that tumoral BRD4 expression is associated with T-bet+ TILs, relatively aggressive clinicopathological features, and a poor disease-free outcome in breast cancer.

## Methods

### Patient cohort

The patient cohort was composed of a prospectively ascertained consecutive series of women with axillary lymph-node negative (ANN) breast cancer, who were enrolled at eight Toronto hospitals from September 1987 to October 1996 as previously described [30, 33]. The clinicopathological features of the cohort have been reported previously [34], and disease-free survival (DFS) and overall survival (OS) data have also been collected with minimum follow-up time of 56 months after surgery and median follow-up time of 100 months. Written informed consent was obtained from all study participants. Approval of the study protocol was obtained from the Research Ethics Board of Mount Sinai Hospital (#01–0313-U) and the University Health Network (#02–0881-C).

### Definition of intrinsic subtypes

Molecular subtypes for tumors were defined based on previous publications [35–37]. HER2 subtype consisted of tumors positive for HER2 overexpression. Luminal subtype included tumors that were negative for HER2 overexpression and positive for ER. Basal subtype included tumors that were negative for HER2 overexpression and ER, and positive for CK5 and/or EGFR. The luminal subtype was subsequently distinguished into luminal A and luminal B based on PgR, p53 status and Ki-67 labeling index. Tumors with a Ki-67 labeling index of ≥14% and were negative for PgR or positive for mutant p53 were assigned to the luminal B subgroup [37].

### Quantitation of T-bet+ TILs using tissue microarrays

Tissue microarrays (TMAs) constructed from formalin-fixed, paraffin-embedded (FFPE) tumor blocks were examined by an expert breast pathologist (AMM) to quantitate for T-bet+ TILs and other immunohistochemical markers as described previously [29].

### Gene expression

Data from gene expression microarray profiling performed previously in our laboratory were statistically analyzed. The mRNA expression profiling was conducted on 19 k arrays (18,981 cDNA/EST clones) manufactured by the University Health Network Microarray Center at the Ontario Cancer Institute (https://www.pmgenomics.ca/arrays/index.htm). Tumor and reference cDNAs (5μg) were indirectly labeled using aminoallyl nucleotide analogs with Cy3 and Cy5 fluorescent tags respectively. Of the 137 flash-frozen ANN tumors analyzed for mRNA expression, 47 tumors had available IHC data for T-bet+ TILs, in which six were T-bet+/high and 41 were T-bet−/low. Supervised statistical analyses and hierarchical clustering were conducted on the gene clones using BRB ArrayTools software (http://linus.nci.nih.gov/BRB-ArrayTools.html).

### Immunohistochemical staining and analysis of BRD4

Immunohistochemical (IHC) staining was performed to examine BRD4 protein expression and localization using polyclonal anti-human BRD4 (HPA061646, Sigma Aldrich) published on the public protein database, The Human Protein Atlas project (https://www.proteinatlas.org/ENSG00000141867-BRD4/antibody). After optimizing the BRD4 antibody for IHC staining on a series of control normal and breast tumor tissues, the BRD4 protein expression was assessed on the TMAs from the previously described cohort of women with ANN breast cancer [30, 33, 34]. The automated BenchMark XT system (Ventana Medical Systems, Inc., Tucson, AZ) was used to perform the IHC staining. The slides were pre-treated with CC1 (Tris-based EDTA buffer, pH 8.0) (Ventana), and incubated with the BRD4 antibody at a 1:300 dilution. Complete pathological report and the level of T-bet+ TILs were available for each tumor in this study.

Immunohistochemically-stained sections were examined for nuclear BRD4 expression, and quantitated using the Allred scoring method [38] by a pathologist with subspecialty training in breast pathology (FT). The score consisted of two components: 1) the average intensity of BRD4 staining (negative: 0; weak: 1; medium: 2; and strong: 3), and 2) the percentage of BRD4-stained nuclei (none: 0; < 1%: 1; 1–10%: 2; 11–33%: 3; 34–66%: 4; and 67–100%: 5). The sum of the two component scores is the overall score with possible values of 0 or 2–8. Due to the lack of validated cut-offs for BRD4 in breast cancer, an arbitrary cut-off score of 6 was decided by assessing nuclear BRD4 expression levels in breast cancer cases that were available in The Human Protein Atlas project.

### Statistical analysis

Genes were ranked based on the fold-difference in expression between T-bet+/high and T-bet−/low tumors as determined by SAM (Significance Analysis of Microarrays) moderated t-test. Chi square test and Fisher exact test were used to analyze the BRD4 marker associations with T-bet TILs, Jagged1, clinicopathologic variables, IHC markers (markers used to define intrinsic subtype), and intrinsic subtype. Clinicopathological variables used in the analyses were selected based on previous studies performed in this cohort [33, 34, 37, 39]. The association of DFS with BRD4 and T-bet marker statuses was examined with log rank test and presented as Kaplan-Meier survival curves.

A *P* value significance criterion of < 0.05 was applied for the tests. Statistical analyses of associations were performed using SAS 9.1 software (SAS Institute, Inc.). Survival curves were plotted using R statistical software, version 2.15.0 (http://r-project.org/).

## Results

### Association of BRD4 mRNA expression in breast cancer with T-bet+ TILs

**Fig. 1:**
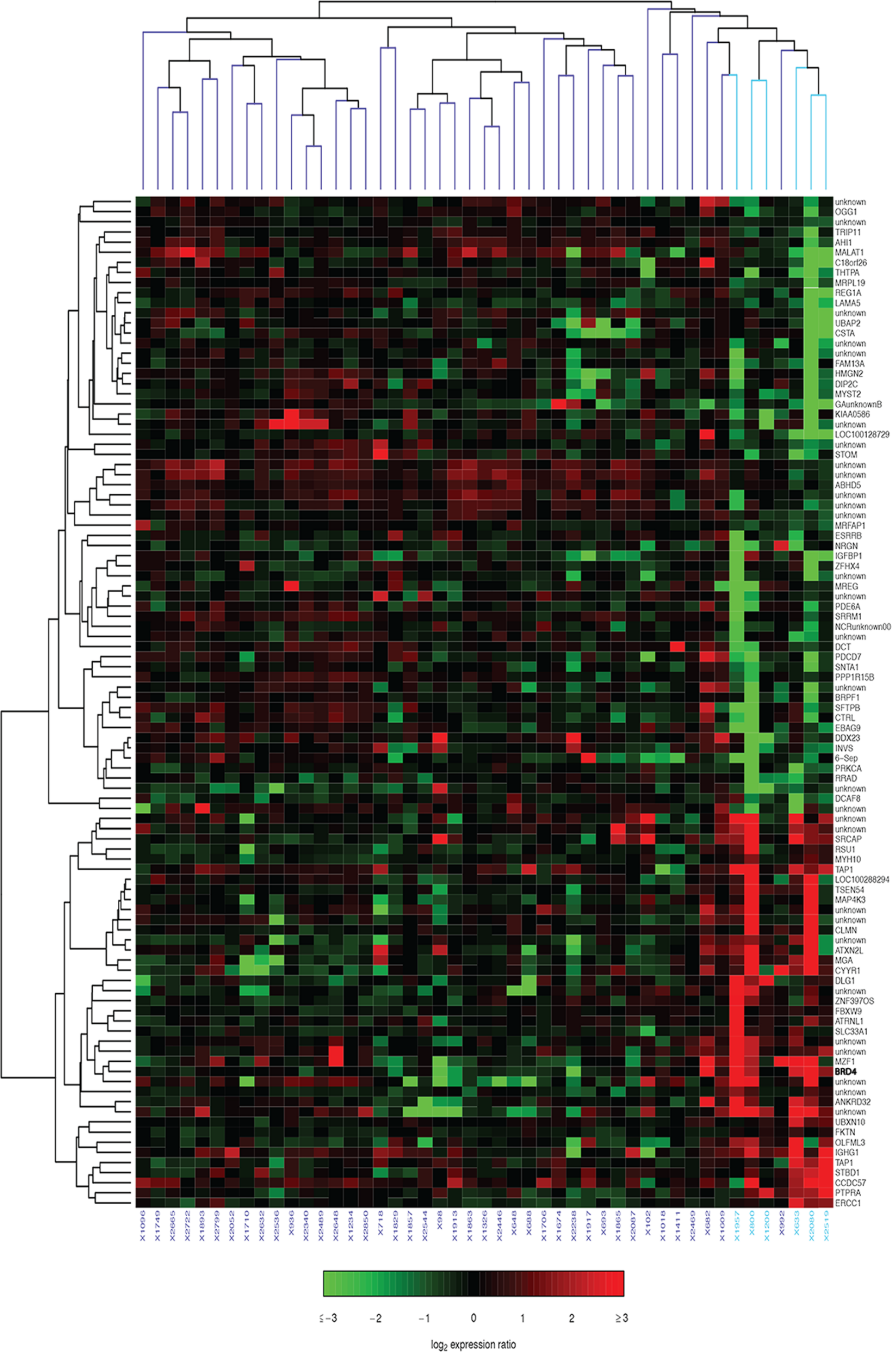
Heat map of top 100 differentially-expressed genes between T-bet+/high (blue) tumors and T-bet−/low tumors (purple)

The mRNA expression differences associated with T-bet+ TIL status were examined by interrogating gene expression microarray data that consisted of 6 T-bet+/high and 41 T-bet−/low breast tumors (Supplementary Material 1 and 2). The top 100 differentially expressed mRNAs (*p* < 0.005) were ranked by Significance of Microarray (SAM), and are presented in a heat map (Fig. 1). One of the top differentially expressed genes associated with T-bet+ TILs (Supplementary Material 3) chosen for further study was BRD4 (*p* = 5.38E-05, FDR = 43.6%), a gene of interest for its potential immune modulatory role in tumors via promotion of NF-kB-mediated inflammation. BRD4 expression in T-bet+/high tumors was up to 44-fold higher than that in T-bet−/low tumors.

### Protein expression and localization of tumoral BRD4

**Fig. 2:**
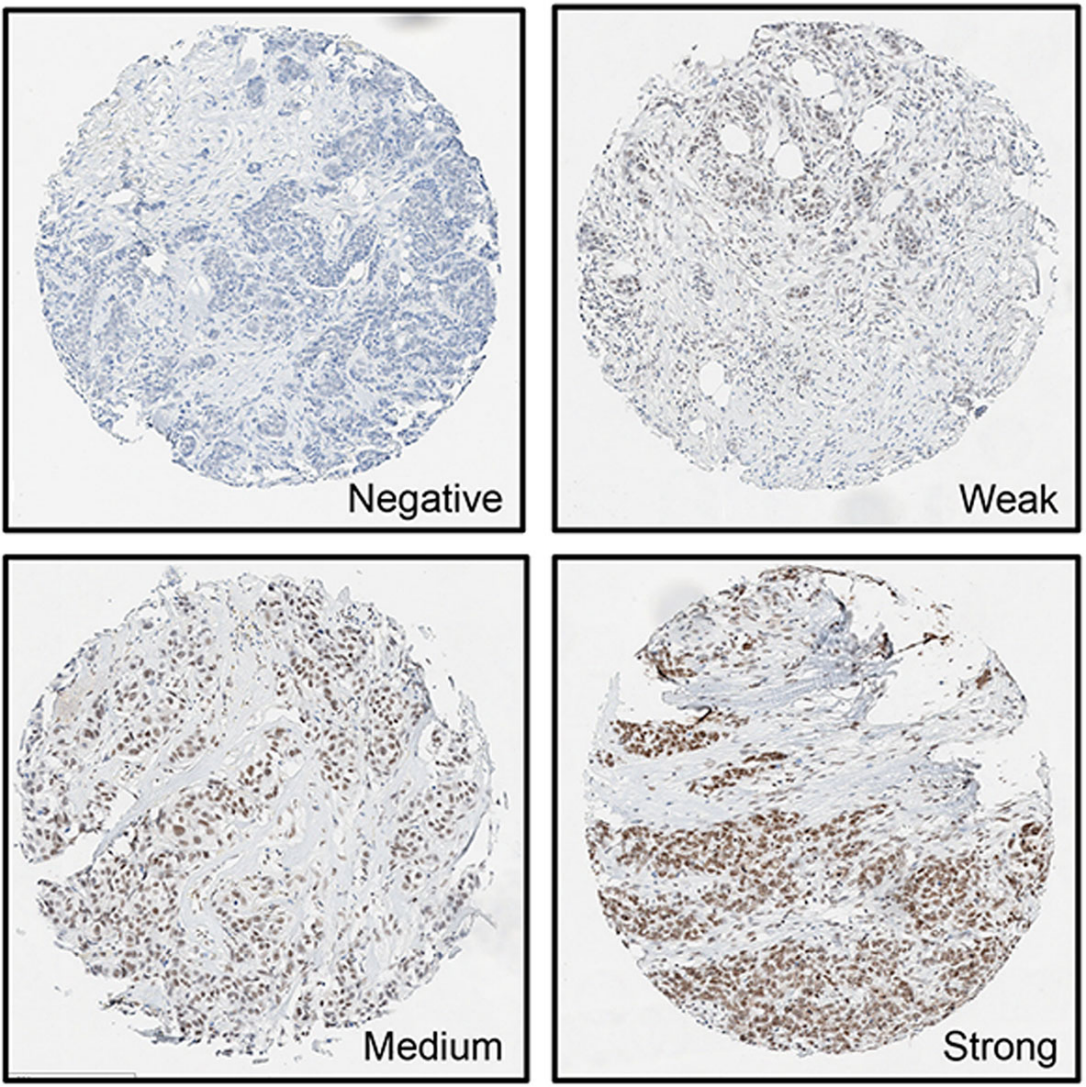
Immunohistochemical intensity of BRD4 in breast tumor TMAs: Negative = 0, Weak = 1, Medium/Moderate = 2, Strong = 3

Immunohistochemistry was performed on TMAs to examine the differential protein expression of BRD4 (Fig. 2). Tumoral BRD4 expression that was assigned an Allred score of 6 or higher was considered to be BRD4 positive in this study. Overall, BRD4 positivity was observed in 76.6% of tumors (*n* = 469/612).

### Association between tumoral BRD4, T-bet+ TILs, and Jagged1

A number of studies have indicated BRD4 to be an upstream regulator of Jagged1 – a ligand that has been shown to participate in various signaling pathways with effects on both intrinsic tumorigenic functions and immune functions. Therefore, we have examined Jagged1 mRNA and protein expression that previously had been quantitated by in situ hybridization (ISH) and IHC respectively in the ANN cohort [40]. BRD4 positive tumors were associated with T-bet+ TILs (*p* = 0.0415) (Table 1), as well as with Jagged1 mRNA (*p* = 0.0171) (Table 2) and protein (*p* = 0.0010) (Table 3) expression. Moreover, Jagged1 mRNA-positive tumors were associated with T-bet+ TILs (*p* = 0.0091) (Table 4).

**Table 1:** Association of tumoral BRD4 expression with T-bet+ TILs

Marker†	BRD4/low	%	BRD4/high	%	*P*-value*
	(*n* = 143)		(*n* = 469)		
	Number		Number		
Tbet+					
Low	78	54.5	317	67.6	0.0415
High	2	1.4	34	7.2	
ND‡	63	44.1	118	25.2	

‡Unknown, not done or missing

*from Fisher’s exact test; ND groups were not used in testing

**Table 2:** Association of tumoral BRD4 expression with Jagged1 mRNA expression

Marker		BRD4/low	%	BRD4/high	%	*P*-value*
		(*n* = 127)		(*n* = 392)		
		Number		Number		
Jagged1 mRNA	Low	58	45.7	133	33.9	0.0171
	High	69	54.3	259	66.1	

*from Chi-Square test

**Table 3:** Association of tumoral BRD4 expression with Jagged1 protein expression

Marker		BRD4/low	%	BRD4/high	%	*P*-value*
		(*n* = 110)		(*n* = 366)		
		Number		Number		
Jagged1 protein	Low	71	64.5	171	46.7	0.0010
	High	39	35.5	195	53.3	

*from Chi-Square test

**Table 4:** Association of tumoral Jagged1 mRNA expression with T-bet+ TILs

Marker	Jagged1/low	%	Jagged1/high	%	*P*-value*
	(*n* = 157)		(*n* = 241)		
	Number		Number		
Tbet+					
Low	151	96.2	214	88.8	0.0091
High	6	3.8	27	11.2	

*from Chi-Square test

### Tumoral BRD4 expression and clinicopathologic and molecular parameters

Tumors exhibiting high levels of BRD4 expression (BRD4+/high) were more likely to be larger (*p* = 0.0049), and were associated with pre-menopausal status (*p* = 0.0018) (Table 5). BRD4+/high tumors were also associated with a high proliferative index as determined by Ki-67 expression (*p* = 0.0009) (Table 6).

**Table 5:** Association of tumoral BRD4 expression with clinicopathologic parameters

Characteristic	BRD4/low	%	BRD4/high	%	*P*-value**
	(*n* = 143)		(*n* = 469)		
	Number		Number		
Number of Recurrences	16	11.2	68	14.5	
Menopausal status					
Pre	30	21.0	172	36.7	0.0018
Peri	6	4.2	22	4.7	
Post	106	74.1	274	58.4	
ND‡	1	0.7	1	0.2	
Lymphatic Invasion					
Yes	19	13.3	56	12.0	0.6592
No	123	86.0	411	87.6	
ND‡	1	0.7	2	0.4	
Tumor Size					
< =0.5 cm	3	2.1	6	1.3	0.0049
> 0.5 to 1 cm	26	18.2	37	7.9	
> 1 to 2 cm	64	44.8	213	45.4	
> 2 to 5 cm	45	31.5	193	41.2	
> 5 cm	4	2.8	19	4.1	
ND‡	1	0.7	1	0.2	
Estrogen receptor					
Positive	87	60.8	302	64.4	0.1249
Negative/Equivocal	28	19.6	108	23.0	
ND‡	28	19.6	59	12.6	
Progesterone receptor					
Positive	80	55.9	267	56.9	0.0935
Negative/Equivocal	35	24.5	143	30.5	
ND‡	28	19.6	59	12.6	
Histological grade					
1^a^	49	34.3	142	30.3	0.1497
2	53	37.1	157	33.5	
3	25	17.5	127	27.1	
ND‡	16	11.2	43	9.2	
Adjuvant treatment					
Hormonal	70	49.0	193	41.2	0.2223
Chemotherapy	16	11.2	82	17.5	
Both	4	2.8	13	2.8	
None	52	36.4	180	38.4	
ND‡	1	0.7	1	0.2	
Age (years)					
Mean	58.25		55.14		
SD	10.13		11.86		
Minimum	33.51		25.49		
Maximum	73.82		75.82		

‡Unknown, not done or missing

**Chi-square test; ND groups were not used in testing

^a^Includes mucinous, lobular and tubular subtypes

**Table 6:** Association of tumoral BRD4 expression with IHC markers

Marker†	BRD4/low	%	BRD4/high	%	*P*-value**
	(*n* = 143)^a^		(*n* = 469)^a^		
	Number		Number		
Her2					
Negative	129	92.8	414	93.2	0.8587
Positive	10	7.2	30	6.8	
ER					
Negative	35	29.2	109	28.1	0.8196
Positive	85	70.8	279	71.9	
PR					
Negative	59	49.6	167	42.2	0.1533
Positive	60	50.4	229	57.8	
EGFR					
Negative	117	96.7	376	93.3	0.1931
Positive	4	3.3	27	6.7	
CK5					
Negative	105	85.4	341	80.4	0.2137
Positive	18	14.6	83	19.6	
Ki67					
< 14%	64	54.7	144	37.4	0.0009
> =14%	53	45.3	241	62.6	

**from Chi-Square or Fisher’s exact test

^a^IHC marker data are not available for some tumors

Complete data to generate molecular subtypes was available for 375 tumors (Table 7). Molecular subtypes did not differ significantly between BRD4+/high and BRD4−/low tumors. However, a trend towards an overall difference among the subtypes was observed.

**Table 7:** Association of tumoral BRD4 expression with intrinsic subtypes

Subgroup	BRD4/low		BRD4/high		*P*-value**
	(*n* = 143)^a^		(*n* = 469)^a^		
	Number	%	Number	%	
Basal	11	13.6	55	18.7	0.068
Her2	8	9.9	25	8.5	
Luminal A	57	70.4	168	57.1	
Luminal B	5	6.1	46	15.7	

**from Chi-Square test

^a^Subtype data are not available for some tumors due to unavailable IHC markers data

### Prognostic relevance of tumoral BRD4 expression in the context of T-bet+ TILs

**Fig. 3:**
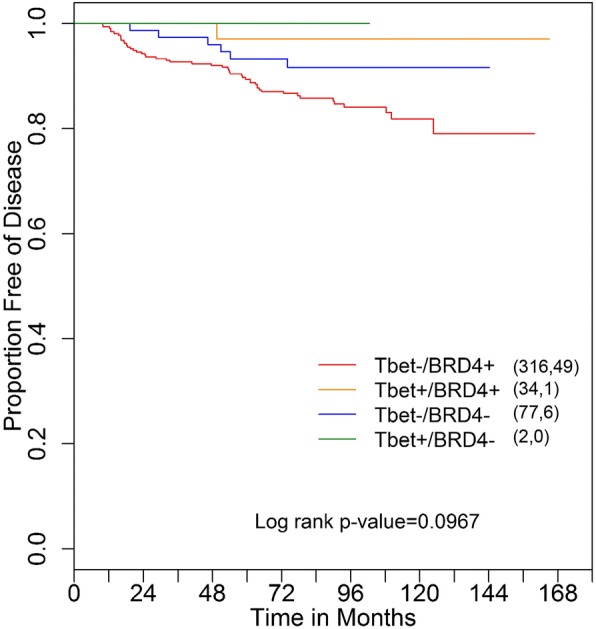
Kaplan-Meier disease-free survival of ANN patients based on BRD4 and T-bet TIL statuses: The first number in the parenthesis denotes the number of patients, and the second number denotes the number of recurrences in the corresponding group

Disease-free survival (DFS) among all four subgroups (T-bet+/high, BRD4+/high; T-bet+/high, BRD4−/low; T-bet−/low, BRD4+/high; T-bet−/low, BRD4−/low) was analyzed. While the overall difference of DFS among the four groups was not significant, T-bet−/low, BRD4+/high trended towards higher recurrence rate than other groups (log rank test *p* = 0.0967) (Fig. 3).

**Fig. 4:**
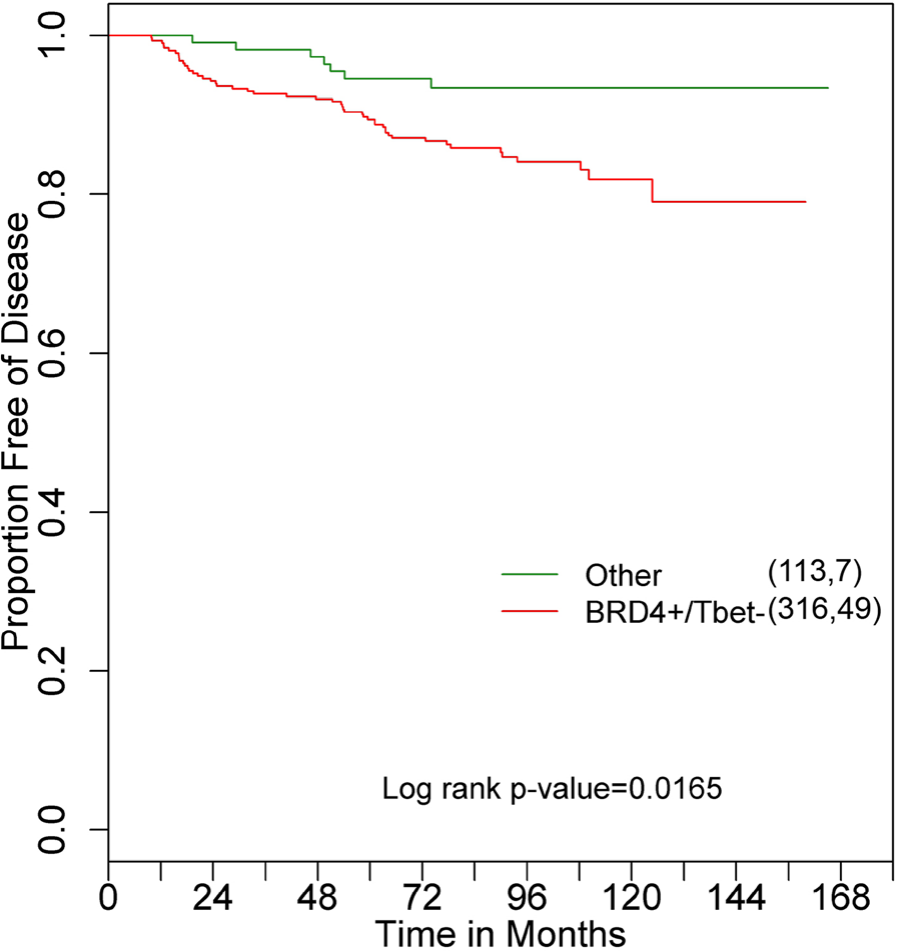
Kaplan-Meier disease-free survival of BRD4+/high, T-bet−/low ANN patients (Red) in comparison to the rest of the subgroups (i.e. T-bet−/low, BRD4−/low; T-bet+/high, BRD4+/high; T-bet+/high, BRD4−/low) (Green): The first number in the parenthesis denotes the number of patients, and the second number denotes the number of recurrences in the corresponding group

Based on this observation, DFS between the T-bet−/low, BRD4+/high group and the combination of other groups was statistically compared, in which patients with T-bet−/low, BRD4+/high tumors were shown to have a significantly a poorer DFS (log rank test *p* = 0.0165) (Fig. 4). Compared to the other subgroups combined, the T-bet−/low, BRD4+/high group was associated with reduced DFS in univariate analysis (LR test *p* = 0.0207, RR = 2.55, 95% CI, 1.15–5.62) (Table 8). This association was retained in multivariate analysis that included traditional clinicopathological parameters and HER2 (LR test *p* = 0.0103, RR = 2.91, 95% CI, 1.29–6.59) (Table 8).

**Table 8:** Results of DFS analysis by Cox proportional hazards model

Prognostic Factor	Univariate				Multivariate			
	RR	95% CI		*P*-value	RR	95% CI		*P*-value
T-bet//BRD4 combinations								
T-bet-/BRD4+ vs.Other	2.55	1.15	5.62	0.0207	2.91	1.29	6.59	0.0103
Her2								
Positive vs. Negative	1.21	0.44	3.36	0.7129	0.51	0.17	1.52	0.2271
Menopausal status								
Pre/Peri vs. Post)	1.08	0.63	1.85	0.7678	0.70	0.25	1.91	0.4806
ER								
Negative/Equi vs. ND/Positive	1.42	0.79	2.53	0.2393	1.36	0.68	2.73	0.3825
Tumor Size								
2–5 cm vs. < 2 cm	2.42	1.39	4.23	0.0018	1.88	1.01	3.50	0.0476
> 5 cm vs. < 2 cm	1.78	0.53	6.05	0.3525	1.33	0.38	4.64	0.6516
Histologic grade								
Grade2–3 vs. Grade1/Subtype^a^	4.23	1.68	10.67	0.0023	3.64	1.41	9.44	0.0078
ND vs. Grade1/Subtype^a^	4.34	1.37	13.81	0.0129	4.41	1.34	14.54	0.0147
Lymphatic invasion								
Present vs. Absent	3.61	2.05	6.33	<.0001	3.96	2.12	7.38	<.0001
Age at diagnosis, yrs								
Linear	0.90	0.70	1.15	0.3858	0.81	0.52	1.25	0.3387
Quadratic	0.90	0.73	1.10	0.2879	0.93	0.75	1.15	0.4801
Adjuvant treatment								
Hormonal vs. None	0.53	0.30	0.93	0.0274	0.51	0.27	0.99	0.046
Chemotherapy vs. None	0.99	0.52	1.88	0.9676	0.53	0.24	1.19	0.1239

^a^Includes mucinous, lobular and tubular subtypes

## Discussion

In this prospectively accrued cohort of women with ANN breast cancer, we examined the relationship between BRD4 and T-bet+ TILs, and evaluated associations of BRD4 expression with Jagged1, clinicopathological features, and clinical outcomes.

We have demonstrated that BRD4 positivity (Allred score of 6 or higher) is significantly associated with T-bet+ TILs, which are a subset of T cells that we have previously determined to be associated with a good outcome in breast cancer patients, despite being associated with adverse clinicopathological features. This suggests a potential link between BRD4-associated tumor progression and the inflammatory lymphocytic infiltrate in breast tumors. BRD4 has been implicated in a number of studies for its role in promoting inflammation [13, 14, 41] notably via activating NF-kB-regulated pathways in cancer cells [17]. NF-kB is a major transcription factor involved in regulating immune and inflammatory responses, and in influencing cancer progression [42, 43]. In particular, NF-kB is crucial in mediating the synthesis of proinflammatory cytokines, such as TNF-α, IL-1, IL-6, and IL-8 [44], which suggests that BRD4 may be an upstream regulator of inflammatory immune response in tumors. Consequently, BRD4 inhibitors, such as JQ1 and I-BET, have been demonstrated to be effective suppressors of inflammation in treating various cancers and inflammatory diseases [13, 14, 41].

Furthermore, BRD4 was associated with pre-menopausal status, large tumor size, and high Ki-67 expression, which are characteristics that are generally associated with a basal subtype. Multiple studies have demonstrated that prognosis of basal breast cancer is positively associated with expression of immune response genes [45–48]. Although no significant overall difference among intrinsic subtypes was observed between BRD4+/high and BRD4−/low tumors, the association of BRD4 expression with features related to the basal subtype reinforces the idea that the association of BRD4 with immunogenic tumors is potentially through its pro-inflammatory functions.

Women with T-bet−/low, BRD4+/high tumors had worse disease-free survival in comparison to the other women. One explanation may lay in the paradoxical roles of inflammation in cancer that is dependent on the immune composition of the tumor. The poor clinical outcome associated with the BRD4+/high group in the absence of T-bet+ TILs suggests that BRD4 may promote tumor progression through upregulation of chronic inflammatory pathways marked by the production of proinflammatory cytokines such as IL-1α, IL-1β, and IL-6. On the other hand, the relatively favorable outcome that is associated with T-bet+/high tumors despite having high BRD4 expression may indicate a dynamic immune interplay, in which the BRD4-mediated production of proinflammatory cytokines in the presence of tumor-specific T-bet+ TILs may reinforce an anti-tumor immune response. The context-specific role of inflammation in tumor development has been previously demonstrated in mouse models of myeloma and B-cell lymphoma [49]. In the latter study, increased local levels of both proinflammatory cytokines (IL-1α, IL-1β and IL-6) and Th1-associated cytokines (INFγ, IL-2 and IL-12) were shown to be consistently correlated with a successful tumor immune response mounted by tumor-specific CD4+ T cells. Hence, in a T-bet+ TIL-mediated tumor microenvironment, BRD4-mediated NF-kB activation, and subsequent proinflammatory cytokine production may contribute to tumor suppression as the pro-inflammatory cytokines have shown to be important in recruiting circulating leukocytes and activating CD4+ T cell functions.

Another explanation may lay in BRD4’s role in the upregulation of Jagged1 expression [2], which was observed to be associated with BRD4 positivity and T-bet+ TILs in this study. Jagged1 is one of the canonical ligands for the Notch receptor family [50, 51] that serves a multifaceted and highly context-dependent function in regular tissue development and cancer progression. The binding of Jagged1 to Notch1 or Notch3 receptors initiates their activation that involves proteolysis by γ-secretase and release of Notch intracellular domain (NICD). NICD translocates to the nucleus and associates with a transcription complex to regulate expression of target genes. In tumors, the paracrine Jagged1-Notch interaction between cancer cells has been shown to promote proliferation, epithelial-mesenchymal transition, angiogenesis, and metastasis [51]. A recent study demonstrated that BRD4 was the upstream regulator of Jagged1 expression and Notch1 signaling, and played an important role in sustaining breast cancer migration and invasion [2]. In patients, BRD4 and Jagged1 expression has been shown to correlate with the presence of distant metastases [2].

Based on the positive associations observed between BRD4 expression, Jagged1 expression, and T-bet+ TILs, Jagged1, through BRD4 regulation, may also be important in mediating tumor-immune cell interaction. Jagged1-mediated activation of Notch signaling has been shown to promote persistence of immature myeloid cells [52] and immunosuppressive IL-10 production [53], which are characteristics possessed by myeloid-deprived suppressor cells (MDSCs). A recent study by Sierra et al. has shown that humanized anti-Jagged1/2 suppressed tumor growth, decreased the accumulation and tolerogenic activity of MDSCs in tumors, and inhibited the expression of immunosuppressive factors, iNOS and arginase, which in turn, promoted CD8+ T cell infiltration into tumors, and improved the in vivo efficacy of T-cell based immunotherapy [54]. Hence, BRD4+/high tumors in the absence of T-bet+ TILs may exhibit BRD4-mediated upregulation of Jagged1 that may induce Jagged-1-Notch1-mediated accumulation and activation of MDSCs, and suppress the infiltration and anti-tumor activity of T-bet+ T cells.

In the presence of T-bet+ TILs, however, Jagged1 may promote anti-tumoral immune response as its expression has shown to be vital in co-stimulation and regulation of Th1 cells through binding of their cell surface receptor, CD46 (membrane cofactor protein, MCP) [55]. The latter study has shown that disturbance of Jagged1-CD46 crosstalk impeded IFNγ secretion in Th1 cells, and CD4+ T cells from patients with Jagged1 mutation (Alagille Syndrome) or CD46 deficiency failed to mount appropriate Th1 responses in vitro and in vivo. This finding, in addition to the positive association between Jagged1 and T-bet+ TILs observed in this study, suggests that in BRD4+/high, T-bet+/high tumors, BRD4-mediated upregulation of Jagged1 may reinforce the anti-tumoral activity of T-bet+ TILs, and facilitate disease-free survival of patients with breast cancer.

## Conclusion

Tumoral BRD4 expression in breast cancer is significantly associated with T-bet+ TILs, clinicopathological features, and a poor disease-free survival in the absence of T-bet+ TILs. On the other hand, the favorable clinical outcome associated with BRD4 expression in tumors with high levels of T-bet+ TILs may reinforce the T-bet+ TIL-driven tumor immune surveillance. The context-specific association of BRD4 expression with disease-free survival based on the presence of T-bet+ TILs suggests that while the anti-inflammatory treatments against cancer, such as BET inhibitors, may be beneficial in reducing chronic inflammation, they may also reduce the tumor-suppressive, T-bet+ TIL-mediated inflammatory immune response. Hence, deeper understanding of BRD4’s immune modulatory roles in different immune contexts may be important in accurately administering BET inhibitors to patients without the risk of dampening the ongoing anti-tumor immune response.

## Abbreviations

ANN: Axillary node-negative
BET: Bromodomain and extra terminal domain
BRD4: Bromodomain-containing protein 4
CK5: Cytokeratin 5
DFS: Disease-free survival
EGFR: Epidermal growth factor receptor
ER: Estrogen receptor
FFPE: Formalin-fixed, paraffin-embedded
HER2: Human epidermal growth factor receptor 2
IFNγ: Interferon-gamma
IL: Interleukin
Ki-67: Marker of proliferation Ki-67
NF-kB: Nuclear factor kappa-light-chain-enhancer of activated B cells
OS: Overall survival
SAM: Significance analysis of microarrays
T-bet: T box transcription factor
Th1: T helper 1
TILs: Tumor-infiltrating lymphocytes
TMAs: Tissue microarrays
TNFα: Tumor necrosis factor-alpha
